# Strength, Stability, and *cis*-Motifs of *In silico* Identified Phloem-Specific Promoters in *Brassica juncea* (L.)

**DOI:** 10.3389/fpls.2016.00457

**Published:** 2016-04-18

**Authors:** Murali Krishna Koramutla, Deepa Bhatt, Manisha Negi, Perumal Venkatachalam, Pradeep K. Jain, Ramcharan Bhattacharya

**Affiliations:** ^1^National Research Centre on Plant Biotechnology, Indian Agricultural Research Institute CampusNew Delhi, India; ^2^Department of Biotechnology, Periyar UniversitySalem, India

**Keywords:** promoter analysis, aphid-resistance, plant-promoters, phloem promoters, transgenic resistance, promoter elements, promoter stability

## Abstract

Aphids, a hemipteran group of insects pose a serious threat to many of the major crop species including *Brassica* oilseeds. Transgenic strategies for developing aphid-resistant plant types necessitate phloem-bound expression of the insecticidal genes. A few known phloem-specific promoters, in spite of tissue-specific activity fail to confer high level gene-expression. Here, we identified seven orthologues of phloem-specific promoters in *B. juncea* (Indian mustard), and experimentally validated their strength of expression in phloem exudates. Significant *cis*-motifs, globally occurring in phloem-specific promoters showed variable distribution frequencies in these putative phloem-specific promoters of *B. juncea*. In RT-qPCR based gene-expression study promoter of *Glutamine synthetase 3A (GS3A)* showed multifold higher activity compared to others, across the different growth stages of *B. juncea* plants. A statistical method employing four softwares was devised for rapidly analysing stability of the promoter-activities across the plant developmental stages. Different statistical softwares ranked these *B. juncea* promoters differently in terms of their stability in promoter-activity. Nevertheless, the consensus in output empirically suggested consistency in promoter-activity of the six *B. juncea* phloem- specific promoters including GS3A. The study identified suitable endogenous promoters for high level and consistent gene-expression in *B. juncea* phloem exudate. The study also demonstrated a rapid method of assessing species-specific strength and stability in expression of the endogenous promoters.

## Introduction

Rapeseed-mustard (*Brassica* spp.) constitute the third most important group of oilseeds in world agriculture. In India, among the three species of rapeseed and mustard Indian-mustard, *Brassica juncea* (L.) is the chief oil-yielding crop. A major threat to productivity of this crop is posed by aphids, a phloem-feeding hemipteran pest (The International Aphid Genomics Consortium, 2010; Bhatia et al., 2011). Lack of resistance-source within the *Brassica* spp. limits the conventional breeding for developing aphid-resistant cultivars therefore, the challenging task of aphid-management solely depends on application of systemic insecticides, which inadvertently enter into the food chain. Transgenic approach by utilizing genes from distant sources seems to be a potential avenue for developing aphid resistance in *B. juncea*. However, the past efforts have led to only moderate success as evident by no commercial release, so far, of any aphid-resistant transgenic plant-type (Dutta et al., 2005; Yu et al., 2014). Whether this bottleneck is due to the lack of effective toxicity of the transgenes or due to its inadequate spatio-temporal expression in the transgenic plants remains enigmatic.

Transgene-expression is governed by the choice of appropriate promoter (Hernandez-Garcia and Finer, 2014). During the early phase of genetic engineering mostly constitutive promoters have been used for a wide range of trait-expression including aphid resistance (Bhatia et al., 2012; Jisha et al., 2015). However, soon it was evident that the constitutive expression of the transgene led to more metabolic pay-offs and often undesirable pleiotropic effects in the transgenic plants (Kasuga et al., 1999; Hsieh et al., 2002; Zhou et al., 2013). Therefore, tailoring transgene-expression with tissue- and temporal-specificity is significant for minimizing such unintended effects of the transgene. In transcriptional control of gene-expression, *cis*-acting promoter elements play a pivotal role (Hernandez-Garcia and Finer, 2014). In many instances, specific DNA-elements in abiotic stress-inducible promoters conferred specificity of gene-expression and helped in mitigating the undesirable effects of constitutive expression (Lee et al., 2003; Kasuga et al., 2004). Alternatively, for restricting transgene-expression in the desired tissues, tissue-specific promoters have been used (Zheng and Baum, 2008). However, lack of understanding the architecture and DNA-motifs of the tissue-specific promoters necessitates more efforts in this direction.

At molecular level, plants respond to aphid-probing by eliciting phloem-based defense (Louis and Shah, 2013). Therefore, phloem-specific promoters have been considered most suitable for transgenic-expression of defense proteins against aphids (Sadeghi et al., 2007; Chakraborti et al., 2009). To that end, several phloem-specific promoters have been isolated from viruses, bacteria and plants; for example, viral promoters (Medberry et al., 1992; Bhattacharyya-Pakrasi et al., 1993; Rohde et al., 1995; Dinant et al., 2004), agrobacterial promoter rolC (Yokoyama et al., 1994), Arabidopsis promoter AtSUC2, and several SUS promoters from diverse species (Truernit and Sauer, 1995; Singer et al., 2011; Dutt et al., 2012). Notwithstanding apparent redundancy in phloem-specific promoters from viruses and bacteria, endogenous plant promoters are preferred for efficient and consistent transgene-expression in plants (Furtado et al., 2008; Hernandez-Garcia et al., 2009).

Availability of genome sequences, microarray database, and transcriptome data has enabled rapid identification of orthologous promoters across the plant species (Lim et al., 2012; Geng et al., 2014). Full-genome array of more and more number of plants has led to the development of several online bioinformatics tools for classification of related sequences and identification of conserved DNA motifs. Analysis tool, such as MEME suit, allows associating these motifs with gene ontology for deriving their functional significance in gene-function relationship (Bailey et al., 2009). Specific activity of the promoters is commonly identified through expression behavior of their cognate genes across the tissues, developmental stages, and environmental conditions (Kasuga et al., 2004; Ruiz-Medrano et al., 2011). Constitutive promoters, despite high level activity, may suffer inconsistency in certain tissues and at certain developmental stages of the plant (Sunilkumar et al., 2002). For example, CaMV 35S is poor in driving transgene-expression during boll formation in cotton, in germ line tissues of *B*. *juncea*, and in dark grown tissues of moss *Physcomitrella patens* (Arumugam et al., 2007; Saidi et al., 2009; Bakhsh et al., 2012). Therefore, in addition to strength, the spatio-temporal stability of activity is crucial in assessing suitability of a promoter for attaining the desired gene-expression.

We report here on *in silico* identification of seven orthologues of phloem-specific promoters in a major oilseed-crop, *B. juncea* and experimental validation of their strength in driving gene expression in the phloem-sap. As structural insight into their architecture, distribution of significant motif-patterns globally associated with phloem-specific promoters were analyzed. In addition, a statistical method based on RT-qPCR data was devised for assessing stability of the promoter-activities over different growth stages of the plant. The study highlights appropriate endogenous promoters for gene-expression in the phloem-sap of *B. juncea*.

## Materials and methods

### Gene ontology analysis

The phloem-specific promoters from diverse plant species were identified based on published literature. The nucleotide sequences of the promoters and their cognate genes were retrieved from NCBI GenBank. Arabidopsis homologs of the promoters and their cognate genes were identified in TAIR database using BlastX programme of NCBI (http://blast.ncbi.nlm.nih.gov/Blast.cgi). Gene ontology analysis of the promoters was performed based on functional classification of their cognate genes. The genes were grouped into functional categories using the GO slim terms from the Arabidopsis information resource annotation (http://www.arabidopsis.org/tools/bulk/go/index.jsp).

### Collection of phloem exudates

Seeds of Indian mustard (*B. juncea)* line Bio-YSR were germinated and grown in 12 inch pots in a net house during the mustard growing season (November-February) of Delhi, India. Since purpose of this study was assessing suitability of the promoters in expressing genes in the phloem-sap, all the gene-expression studies were carried out in the phloem exudates. Phloem exudates were collected at three different growth stages of the *B. juncea* plants viz. vegetative stage (15 days old), bud initiation stage (30 days old) and flowering stage (45 days old) as described in Buhtz et al. (2008). Fully expanded third to sixth leaves from the apical top were excised using a sharp razor blade and immediately transferred into glass tubes containing 20 mM K_2_-EDTA solution. After incubation for 30 min and removal of the initial exudates, the petiole ends were freshly cut to avoid sieve tube embolism. The cut ends were dipped in 500 μl freshly prepared 20 mM K_2_-EDTA solution containing 100 U/ml RNase inhibitor (cat# 1B1410, Ameresco, USA) and left for overnight in a pre-humid plexi-glass box. The phloem exudates collected in the EDTA buffer were frozen in liquid N_2_ and stored at −80°C until used for RNA extraction.

### RNA isolation and cDNA synthesis

Total RNA was isolated using Trizol reagent (cat# 15596029, Invitrogen, USA) according to the manufacturer's instruction. The residual DNA was removed by RNase-free DNase (cat# 79254, Qiagen, USA) according to manufacturer's specification. Yield and purity of RNA was determined by NanoDrop ND-1000 spectrophotometer (Nanodrop technologies, USA). RNA samples with an absorbance ratio OD 260/280 between 1.9 and 2.2 and OD 260/230 greater than 2.0 were used for experimentations. RNA-integrity was verified through gel electrophoresis, by resolving the samples on 1.8% agarose-gel in 1X TAE. First stand cDNA was synthesized from 2 μg of total RNA using a cDNA synthesis kit (cat# 6110A, Takara Bio Inc., Japan), diluted 20 times with nuclease free water and used for PCR or RT-qPCR.

### Primers and RT-PCR

Gene specific primers were designed based on *Brassica* specific homologous sequences using IDT primer quest software (http://www.idtdna.com) (Tables S1, S2). Primers' specificity was examined by validating the desired amplifications in RT-PCR as well as by sequencing of the amplicons. GeneRuler 100 bp Plus DNA Ladder (Cat# SM0323; Thermo Fisher Scientific, USA) was used as size marker in agarose gel. For determining amplification efficiency (E) of the primers in RT-qPCR, a serial tenfold dilution (1–1000) of the cDNA pool was used. A standard graph based on their Ct values was generated with linear regression and the slope using Microsoft Excel. The amplification efficiency (E) for each gene-specific primer was calculated according to the equation: *E* (%) = (10^−1∕*slope*^−1) × 100%. All the RT-qPCR reactions were performed using SYBR green detection chemistry, in a StepOne plus Real time PCR machine (Applied Biosystems, USA). A reaction cocktail of 20 μl was constituted by mixing 2 μl diluted cDNA, 10 μl 2X SYBR Premix Ex Taq II (cat# RR820A, Takara Bio Inc, Japan), 0.4 μl of ROX reference dye and 0.4 μl each of the forward and reverse primers. PCR cycling was carried out at an initial denaturation of 1 m at 95°C, followed by 40 repeated cycles each consists of 95°C for 10 s, 60°C for 30 s, and 72°C for 30 s. To check amplification-specificity, dissociation curve analysis was carried out by constant increase of temperature between 60 and 95°C. All RT-qPCR and the RT-PCR experiments were carried out in three and two biological replicates, respectively with three technical replicates each time. Glyceraldehyde-3-phosphate-dehydrogenase (*GAPDH*) was used as reference gene (Chandna et al., 2012; Bhogale et al., 2014).

### Identification of significant *cis*-elements in phloem-specific promoters

Over represented motifs were identified using MEME (Multiple Expectation Maximization for Motif Elicitation) suite (Bailey et al., 2009), Oligo-analyzer, and info-gibbs programs of RSAT (Regulatory Sequence Analysis Tools) motif discovery tool. The consensus motifs were identified with the following settings: 6–15 bases width, one or more occurrence per sequence, and *E* < 0.01 for only in the given strand of input sequences. Sequences were also aligned using the AlignACE program (http://atlas.med.harvard.edu/cgi-bin/alignace.pl), and the motifs with the highest MAP scores were selected and their web logo was plotted using WebLogo tool (http://weblogo.berkeley.edu/logo.cgi). The discovered motifs were further analyzed by homology search in PLACE and STAMP (http://www.benoslab.pitt.edu/stamp) programs to identify their putative functions. Statistically significant motifs were searched in individual promoters by using FIMO program of MEME with *p* < 0.0001.

### Statistical methods and expression-stability analysis

RT-qPCR data was analyzed by Microsoft Excel based software tools geNorm (Vandesompele et al., 2002), NormFinder (Andersen et al., 2004), BestKeepr (version1) (Pfaffl et al., 2004), and the delta Ct methods (Silver et al., 2006) for ranking the expression-stability of the cognate genes. The geNorm algorithm calculates the gene-stability measure (*M*) among the genes in a given set of samples based on the principle that the logarithmically transformed expression ratios between any two genes should be invariable if both the genes are expressed equally in a given set of samples. It determines rank order of the genes in a set of samples by calculating expression-stability measure (*M*)-values based on geometric averaging of multiple genes as well as pair-wise comparison and stepwise exclusion of the genes from other samples. The *M*-value is inversely proportional to expression-stability of the gene; lower the *M*-value, higher is the expression-stability. *M*-value <1.5 is recommended to identify stably expressed genes. NormFinder calculates expression-stability of the genes in all the samples in any number of groups based on intra- and inter-group variations and combines these values to provide gene-rank depending on the variation in gene-expression. Gene with lower value signifies more stable expression. The Bestkeeper software uses geometric mean of Ct values with a standard deviation (SD) and PCR efficiency (*E*) to determine the best suited standards and combines them into an index by the coefficient of determination and the *P*-value. A lower index score refers to the higher stability in gene expression. The delta Ct method compares relative expression of genes in a pair-wise manner, in which each of the genes is considered as reference gene and compared with the remaining.

## Results

### Identification of homologous phloem-specific promoters in *Brassica juncea*

**Figure 1:**
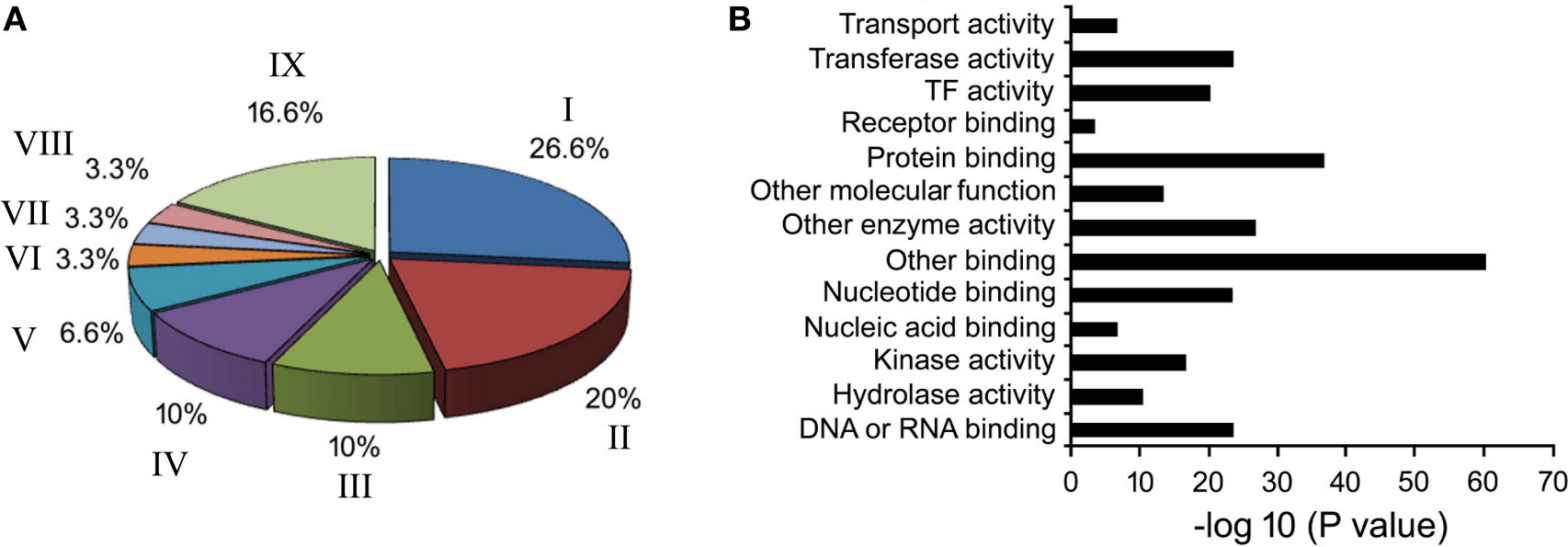
**Gene ontology of phloem-specific transcripts. (A)** Nine functional categories of the transcripts are represented by *different letters in the pie chart. I*. Response to biotic and abiotic stimulus, *II*. Developmental processes, *III*. Transcription, *IV*. Transport, *V*. Signal transduction, *VI*. Electron transport, *VII*. Cell organization and biogenesis, *VIII*. Protein metabolism, and *IX*. Unknown biological processes. **(B)** Distribution of Go terms based on their molecular function.

Based on literature, 39 known phloem-specific promoters were chosen and their sequences were downloaded from NCBI database (Table S3). These promoters, from monocot as well as dicot plants, ranged from smallest AtGLP13-promoter of 762 bp to CmGAS1-promoter of 3000 bp. Based on putative function of their cognate genes, the promoters were classified in two GO terms viz. biological process (Figure 1A) and molecular function (Figure 1B). In biological process among the major categories, about 26.6% of the promoters were found to be stress responsive and grouped under response to stresses, followed by 20%, that were associated with developmental processes, and 10% each controlling genes related to transport and transcription processes. Across the ontogenic groups, seven promoters that were associated with host-response against insects and pathogens, either directly or indirectly, were identified (Table 1). Homologues of the seven promoters were predicted in *Brassica* spp. and identified either in *B. rapa* or in *B. juncea*.

**Table 1:** **Cognate genes of the ***in silico*** identified phloem-specific promoters in ***Brassica juncea*****.

**Gene**	**GenBank Acc no**.	**Function**	**Activity**	**References**
*GLP13*	AC189540.1	GLPs, including superoxide dismutase and oxalate oxidase, are encoded by multi gene families and have diverse enzyme functions	GLPs act as defense related proteins induced by pathogens, insect feeding, and plant hormones viz. MJ, SA, and ET	Dunwell et al., 2008
*GS3A*	U28924.1	Glutamine synthetase (GS) catalyzes the ATP-dependent addition of ammonium (${\text{NH}}_{4}^{+}$) to the γ-carboxyl group of glutamate to produce glutamine, in ammonia assimilation	Involved in nitrogen metabolism, over expression leads to resistance to herbicide Basta; activated by plant hormones MJ, SA and ET, pathogens and aphids	Divol et al., 2005; Pageau et al., 2006
*TGG1*	AY014960.1	Myrosinase cleaves the thio-linked glucose of glucosinolates by hydrolysis	Mediate plant defense to insect herbivores in Brassicaceae members	Rask et al., 2000
*GAS1*	FJ407183.1	GolS1 is a key enzyme in the synthesis of oligosaccharides of raffinose family and galactosylates myo-inositol to form O-alpha D-galactopyranosyl-[1 1]-L-myo-inositol.	Involved in jasmonate mediated resistance to pathogen in Arabidopsis; reduction in aphid-fecundity; highly induced by drought and salt stresses	Cho et al., 2010; Cao et al., 2013
*SUC 2*	EU570076.1	SUC2 transports sucrose from source tissue to sink tissue in Arabidopsis	Highly expressed in phloem companion cells of matured leaves and involved in phloem loading; induced by aphid-infestation in Arabidopsis at early stage	Srivastava et al., 2008; Dubey et al., 2013
*SULTR2*	AJ223495.1	Involved in sulfate transport	Low affinity transporter mainly expressed in vascular tissue; induced by fungal pathogen *Verticillium* in phloem tissues	Takahashi et al., 2000; Howarth et al., 2003
*PP2*	NM_118104.4	PP2 is a poly-GlcNAc-binding lectin	A phloem lectin activated by fungal pathogen and ethylene; over expression of PP2 led to resistance to *Myzus persicae* and English grain aphid in Arabidopsis and wheat, respectively	Zhang et al., 2011; Lee et al., 2014; Fu et al., 2014

### Functional validation of the predicted promoters

**Figure 2:**
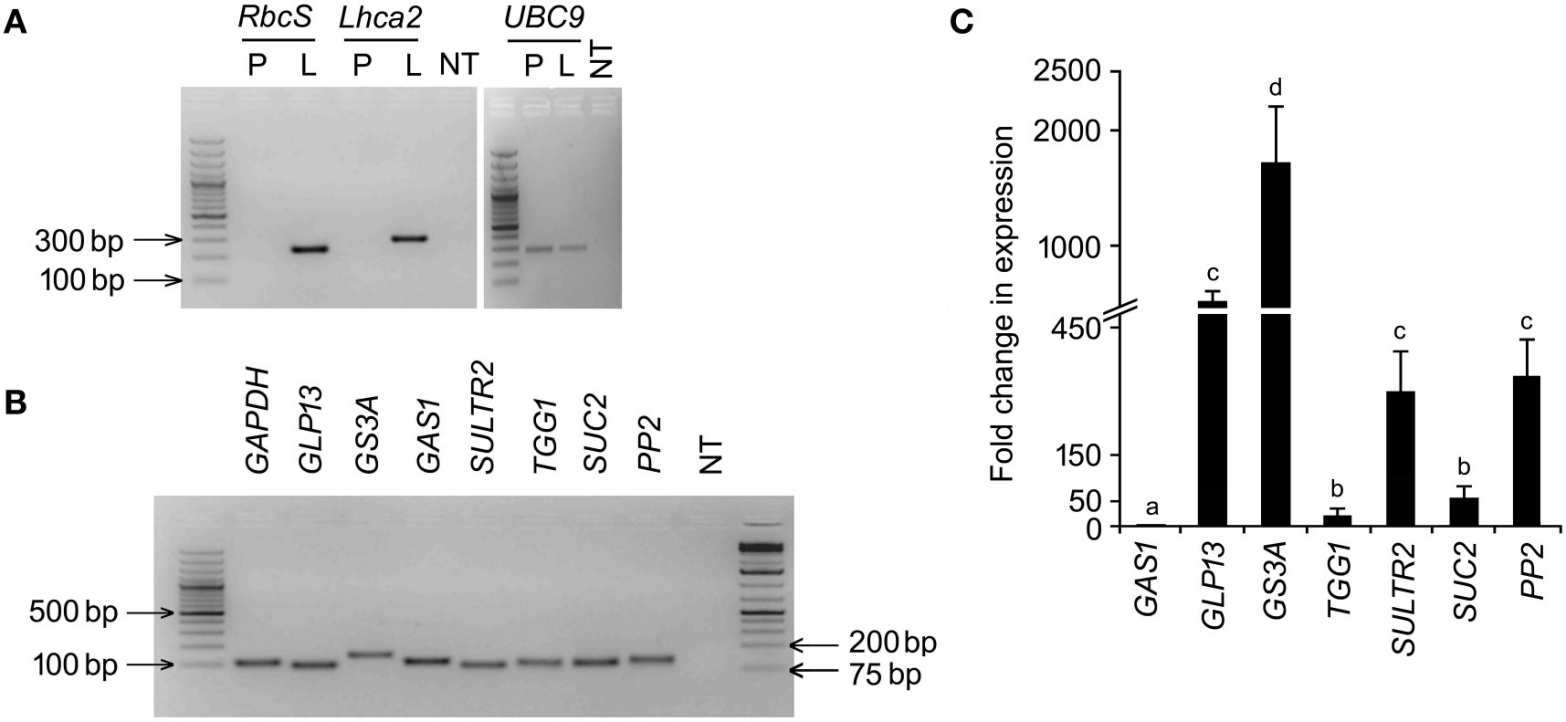
**RT-PCR analysis for the cognate-transcripts of the phloem-specific promoters**. Total RNA was isolated from phloem exudates collected from 45 days old *B. juncea* leaves and assayed for the cognate-transcripts of the *in silico* identified *B. juncea* phloem-specific promoters by RT-PCR and RT-qPCR. **(A)** RT-PCR amplification of *RbcS, Lhca2* and *UBC9* in phloem exudates (P), and leaf tissues (L) along with non-template (NT) control. **(B)** RT-PCR amplification of the cognate-transcripts in phloem exudates. **(C)** RT-qPCR based analysis of the cognate-transcript levels in phloem exudates.

RT-PCR based detection of the cognate mRNAs in the phloem exudates of *B. juncea* and their RT-qPCR based quantitative analysis validated activity of the *in silico* identified promoters and their relative strength, respectively in the phloem exudates of *B. juncea*. Limited availability of sequence information on *B. juncea* genome in public domain database led us to retrieve the cognate gene sequences from other species of *Brassica*. For each of the target transcripts, PCR and RT-qPCR conditions were optimized so that the gene specific primer-pairs amplified single PCR product of desired size and single peak in melt curve analysis (Figure S1). The amplicons were further validated through sequencing. The standard curve analysis for RT-qPCR efficiency revealed a linear regression *R*^2^ of all the primer-pairs ranging between 0.999 and 1.000 and the efficiency ranging from 93 to 105% (Figure S2). Phloem exudates were collected from excised petioles of *B. juncea* plants. The RNAs isolated from the phloem exudates were assayed for the presence of any non-vascular cellular contamination. For that, the RNAs were analyzed for the presence of *RbcS* and *Lhca2* specific amplification in RT-PCR. *RbcS* and *Lhca2* encode two highly abundant photosynthetic proteins rubisco small subunit and chlorophyll a/b binding protein, respectively. *RbcS* and *Lhca2* gene expression is excluded from vascular cells (Sawchuk et al., 2008) and therefore, their mRNAs, despite showing different dynamics during leaf development, are likely to be completely absent in phloem exudates (Buhtz et al., 2008). No amplification of either *RbcS* or *Lhca2* transcripts in RT-PCR unambiguously indicated purity of the phloem exudates (Figure 2A). On the other hand, specific amplification of the cognate genes in the same cDNA sample indicated phloem-bound activity of the seven orthologous *B. juncea* promoters (Figure 2B). The relative transcript levels of the cognate genes were estimated in RT-qPCR analysis and expressed as multifold ratio of their normalized level to the least abundant transcript level of *GAS1* (Figure 2C). The results empirically showed significant variation among the transcript levels indicating significant differences in strength of the promoters. Amongst the variability, *GS3A* showed the highest transcript level followed by *PP2, GLP13* and *SULTR2*. However, quantitative expressions of *PP2, GLP13* and *SULTR2* were statistically similar. Co-amplification of *UBC9* in the exudate-cDNA indicated integrity and optimum level of cDNA in the reactions.

### Identification of signature *cis*-elements in phloem-specific promoters

**Figure 3:**
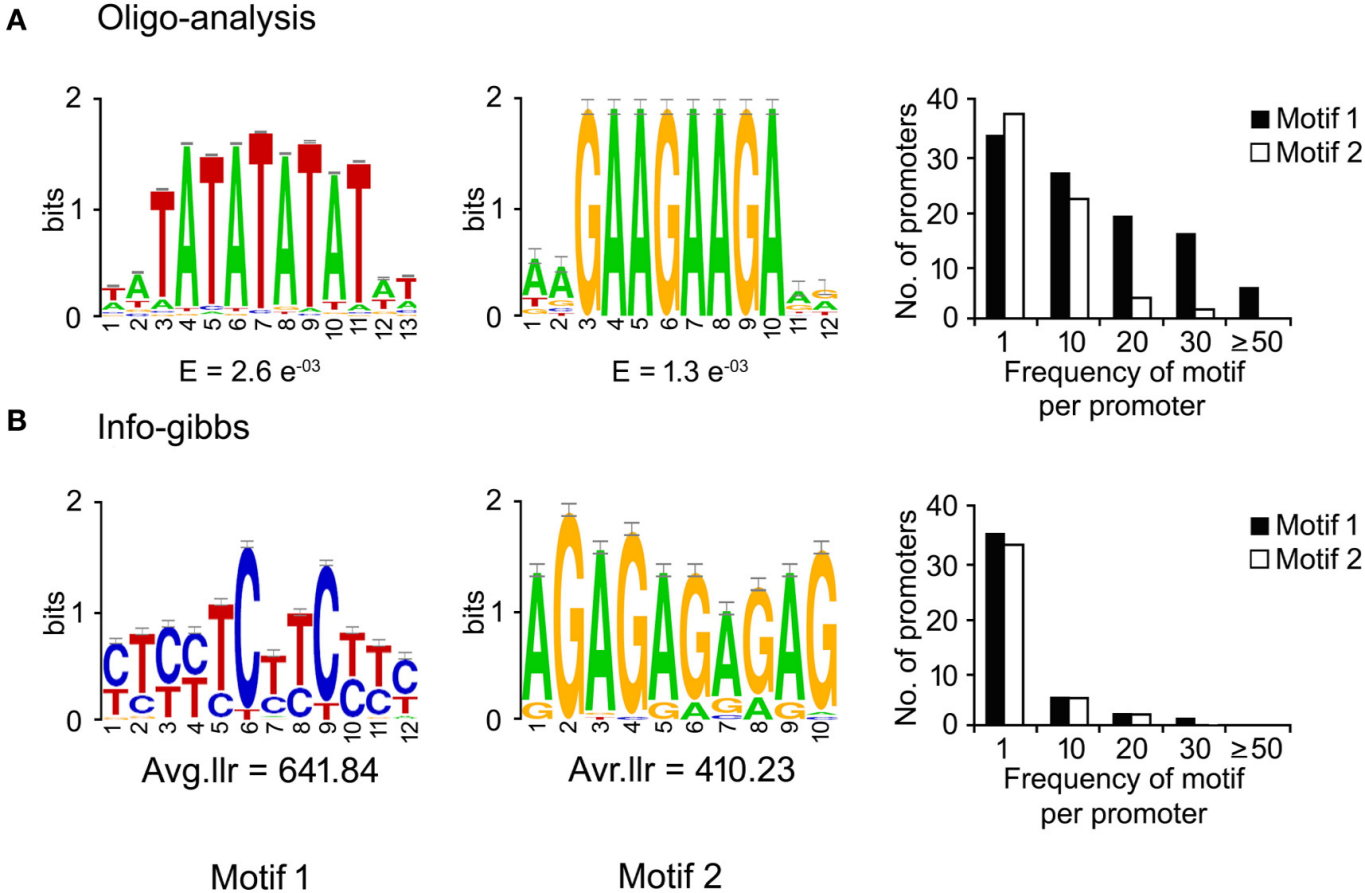
**Significant ***cis***-motifs identified by RSAT oligo-analyzer and info-gibbs**. The motifs were identified by RSAT oligo-analyzer **(A)** and info-gibbs **(B)** motif discovery tool. Frequency distribution of significant motifs were searched by using FIMO program of MEME with *p* < 0.0001. Two motifs showing lowest expectation (*E*)-values and high log likelihood ratio (Avg.llr) in each case and their frequencies in the phloem-specific promoters have been shown. The *X*- and *Y*-axis show the position of nucleotides and the bits score, respectively.

**Figure 4:**
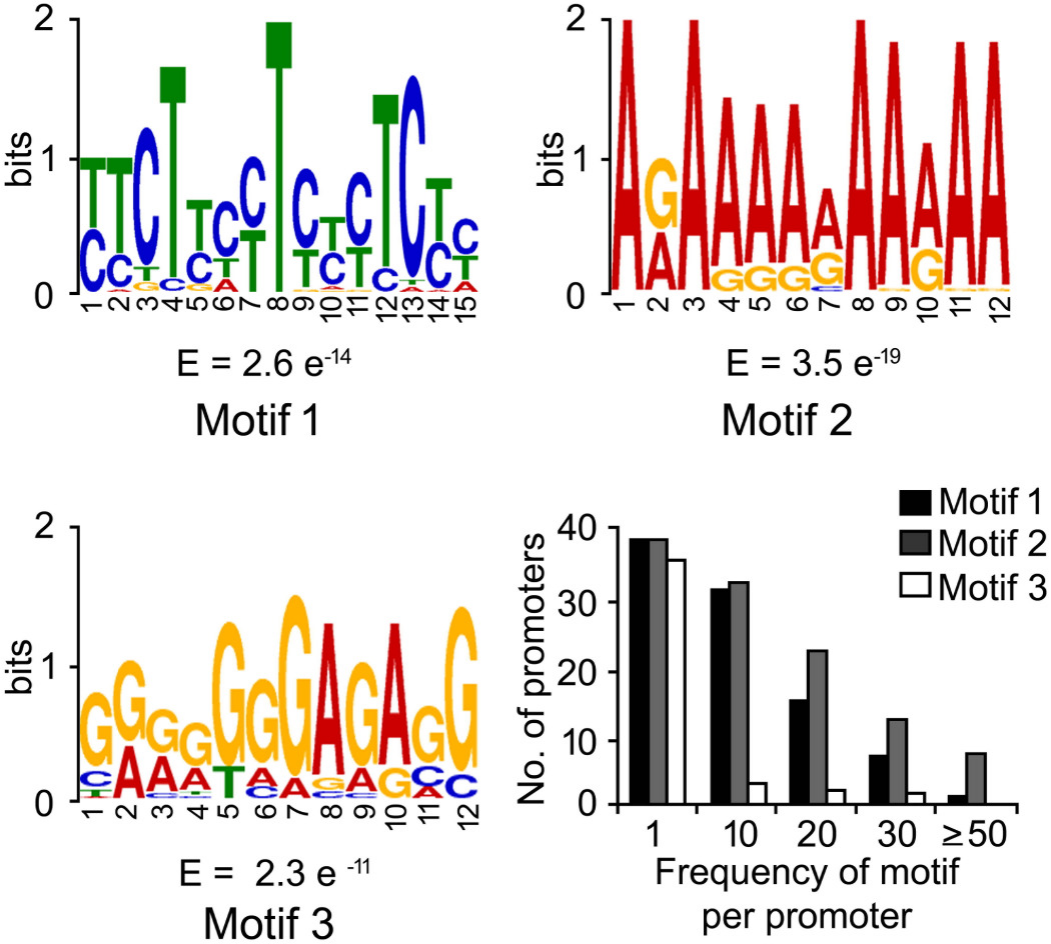
**Motifs with lowest expectation (***E***)-value, identified by MEME suit**. The signature motifs across the phloem-specific promoters were identified by MEME suit. Three motifs showing lowest expectation (*E*)-values and their frequencies are shown. The *X*- and *Y*-axis show the position of nucleotides and the bits score, respectively.

DNA-motifs globally associated with phloem-specific promoters were not known. Therefore, discovery of such motifs was mandatory for understanding the architecture of the putative phloem-specific promoters identified in *B. juncea*. For that, comprehensive set of 39 promoter sequences from diverse origin (described in Table S3) were analyzed, and the over-represented motifs in them were discovered through string based (oligo-analysis of RSAT) and position weight matrix-based (info-gibbs of RSAT, AlignACE, and MEME) motif discovery programmes (Van Helden et al., 1998; Hughes et al., 2000; Defrance and van Helden, 2009). Oligo-analysis revealed the commonly occurring over-represented motifs in all the promoter sequences. Among the identified motifs, the two with lowest expectation (*E*)-values were scored as significant and their frequency of occurrence per promoter has been shown in Figure 3A. The two motifs showed similarity with known plant *cis*-regulatory elements which are responsive to plant hormones, such as auxin and salycilic acid. Independent analysis based on info-gibbs identified a CT-rich signature motif (Figure 3B) specifically present in the promoters of phloem-specific transcripts. Info-gibbs predicted another AG-rich motif showing similarity to CTRMCAMV 35S motif which was found in -60 nt downstream of transcription start site of the CaMV 35S RNA and known to enhance gene expression driven by the CaMV 35S (Pauli et al., 2004). Analysis based on MEME predicted three significant motifs with low (*E*)-values that have been shown in Figure 4. Among the three, two motifs were grouped in CT/GA-rich repeat motifs specific to phloem-specific promoters. The other one showed similarity with 314MOTIFZMSBE1 which is a positive *cis*-element located between –314 and –295 region of maize *Sbe1* promoter and required for high level as well as sugar responsive expression. The AlignACE programme was used to identify two A/T rich degenerate motifs in phloem-specific promoter sequences which are widespread in eukaryotic genomes (Figure S3). Use of multiple motif discovery programmes led to the identification of nine significant signature *cis*-elements specific to the phloem-specific promoters across the vascular plant species.

### Signature *cis*-elements in *B. juncea* phloem-specific promoters

**Figure 5:**
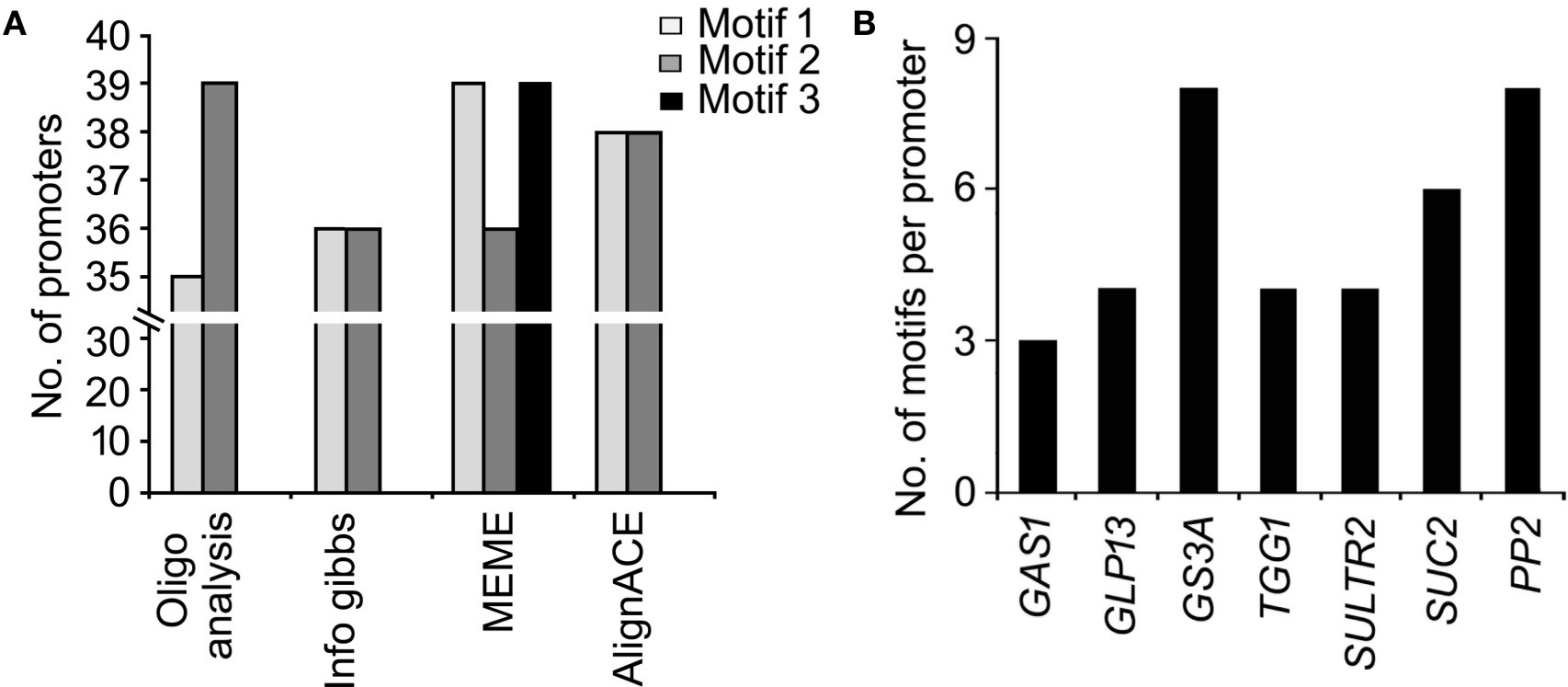
**Significant ***cis***-elements globally associated with phloem-specific promoters and their frequency distribution in ***B. juncea*** promoters**. **(A)** Global occurrence of nine signature *cis*-elemnts in phloem-specific promoters of diverse vascular plants. **(B)** Frequency distribution of signature *cis*- elements in the seven phloem-specific promoters of *B. juncea*. Frequency distribution of significant motifs were searched by using FIMO program of MEME with *p* < 0.0001.

The global frequency of the nine identified signature *cis*-elements (Figure 5A) and their distribution in the seven putative phloem-specific promoters of *B. juncea* were analyzed (Figure 5B). The *B. juncea* promoters showed occurrence of all the signature *cis*-elements, albeit their distribution and frequency on each promoter varied (Figure 5B). Promoters of *GS3A* and *PP2* contained eight of the nine signature motifs followed by promoter of *SUC2* with six motifs. Promoters of *GLP13, TGG1*, and *SULTR2* each contained four of the nine motifs. However, no apparent correlation between the frequency of the *cis*-elements and the relative strength of the promoters could be established. For example, PP2 promoter in spite of containing equal number of the motif elements as GS3A generated much lesser transcript level compared to GS3A. Interestingly, a notable difference between the GS3A and PP2 promoter was exclusive presence of a degenerate motif (GRRRGGGAGASG; *R* = A/G, S=G/C), identified in MEME based analysis, only in case of GS3A promoter. However, further experimentation is warranted before attributing this degenerate motif to high expression of *GS3A*. Nevertheless, the results of RT-qPCR analysis validated the applicability of the identified signature elements in identifying the phloem-specific promoters.

### Stability analysis of promoter activity

**Figure 6:**
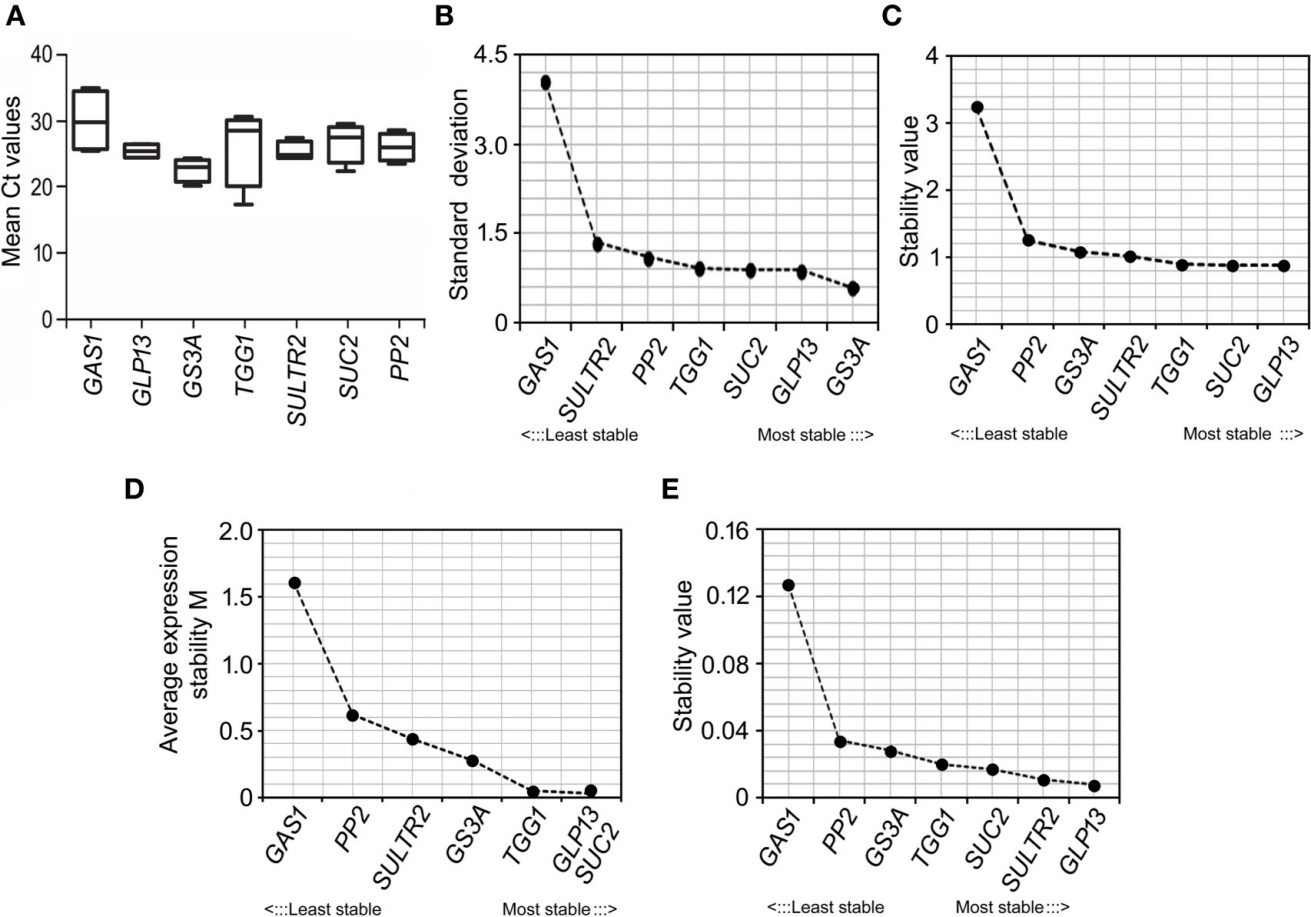
**RT-qPCR based analysis of expression-stability of the ***B. juncea*** phloem-specific promoters. (A)** Mean Ct values of the cognate-transcripts analyzed by RT-qPCR in phloem-cDNA samples collected at different growth stages of *B. juncea* plants. **(B–E)** Ranking of the *B. juncea* phloem-specific promoters in terms of expression-stability measured by four Excel based statistical methods, BestKeeper **(B)**, deltaCt method **(C)**, geNorm **(D)**, and NormFinder **(E)** and plotted in increasing manner from left to right.

Promoter-activity of the *in silico* identified *B. juncea* promoters at different growth stages of the plant were analyzed by assessing their cognate-transcript levels at the vegetative, bud initiation and flowering stage. RT-qPCR analysis of the cognate-transcripts captured at different growth stages empirically showed likely influence of plant developmental stages on the activity of these promoters (Figure 6A). Based on mean Ct values, *GS3A* showed highest average transcript levels, followed by *GLP13* and *PP2*, across the developmental stages of *B. juncea* plants. Independent analysis of the cognate-transcript levels within the vegetative and reproductive stage also showed the highest relative expression of *GS3A* at both the growth-stages. However, in general the transcript levels of all the cognate genes were consistently lower during the reproductive stage compared to their levels at the vegetative stage, and also the expressions were further reduced as the plants grew toward maturity.

Irrespective of strength, the consistency in promoter-activity across different growth stages was compared in mathematical terms. For that the gene-expression data were analyzed by four Excel based statistical methods, BestKeeper, deltaCt method, geNorm, and NormFinder. In these methods the transcript stability was measured as a function of standard deviation in transcript abundance across the samples. Based on stability of their cognate-transcripts, the promoters were ranked by stepwise exclusion of one at a time with lowest stability. The output of the individual statistical method indicated different ranking pattern of the promoters in terms of consistent activity (Figures 6B–E). The analysis by BestKeeper empirically showed *GS3A* as the most stably expressed whereas, the other three methods ranked *GLP13* to be the most stable across the growth stages. In contrary, irrespective of statistical methods *GAS1* was ranked as the least stable in expression. In geNorm based analysis, the *M*-values of the six promoters except GAS1 promoter were <1.5 and therefore they were categorized as stable promoters (Vandesompele et al., 2002). The apparent variation in ranking within the six promoters, depending on the statistical method used, was insignificant. In spite of differential ranking pattern, all the methods unambiguously indicated higher stability in promoter activity in case of all the *B. juncea* promoters except GAS1.

## Discussion

Crop losses due to aphids are estimated to be hundreds of millions of dollars annually (Blackman and Eastop, 1984; Morrison and Peairs, 1998). In Indian mustard (*B. juncea*), aphid-resistant germplasm has been largely unavailable (Bhatia et al., 2011). Therefore, transgenic development for aphid resistance has been given significant priority (The International Aphid Genomics Consortium, 2010). Past efforts on transgenic development utilized mostly the constitutive promoter CaMV 35S in transgene-expression (Sadeghi et al., 2007; Nakasu et al., 2014). Use of CaMV 35S promoter raised several concerns (Ho et al., 1999; Oraby et al., 2014). Moreover, since aphids feed exclusively the phloem-sap use of phloem-specific promoters is more appropriate for the expression of aphid-deterrent genes.

The phloem-expressing promoters play significant roles in many plant processes including organ development, sink-source flow of photoassimilates etc. Gene ontology of the phloem-expressing genes classified them in nine groups depending on their cellular functions. Primarily based on defense associated function we narrowed down to seven of them and identified their homologs in *B. juncea* (Table 1). Validation of the promoter-function and strength traditionally requires development of large number of transgenics with single copy insertion of the promoter-reporter cassette. Being tedious, such approach limits the number of recruited promoters in such studies (Dutt et al., 2012). Therefore, for verifying the phloem-bound activity and assessing the strength of the *in silico* identified *B. juncea* promoters, we resorted into RT-qPCR based analysis of their cognate-transcripts in the phloem exudates. Phloem exudate instead of phloem and companion cells *per se* were more relevant so as to analyse the promoters in the context of expressing aphid deterrent genes in the phloem-saps.

Collection of phloem exudates is complicated in most species because of the normal occlusion response of the sieve tube upon wounding (Guelette et al., 2012). To prevent this, *B. juncea* petioles were washed with EDTA immediately upon excision and prior to the collection of exudates. EDTA chelates Ca^2+^ ions and inhibits reactions leading to blockage of sieve pores (Knoblauch et al., 2001). In experimental validation, all the seven promoters showed activity in the phloem-sap, though at variable strength, as evident from their cognate-transcripts in the phloem exudates. Relative abundance of the cognate-transcripts, which is by far governed by the strength of their promoters, showed three distinct levels of gene-expression. Interestingly, GS3A promoter conferred significantly higher gene-expression compared to the second level of expression shown by PP2, GLP13 and SULTR2, and the least gene-expression by TGG1, SUC2, and GAS1. Strength of the promoters depends on the presence of specific DNA elements in and around it (Hernandez-Garcia and Finer, 2014). Therefore, further studies on significant *cis*-motifs in these promoters were warranted.

Though, a large number of promoters with activity in the phloem-have been isolated previously, only in case of few the specific *cis*-elements were proposed (Schneidereit et al., 2008). Therefore, discovery of the major *cis*-motifs globally associated with the phloem-specific promoters was a prerequisite for structural insight into the *B. juncea* promoters. Further it was necessary to perform the bioinformatic analysis preferably on a larger and more comprehensive set of promoter sequences retrieved from across the vascular species. Analysis of 39 diverse phloem-specific promoters identified the core promoter elements in addition to other potentially important *cis*-regulatory elements responsive to hormones, light and stress related cues (data not shown). Frequent occurrence of such elements clearly corroborated influence of most of the biotic and environmental stresses on the vascular gene-expression. For example, phloem-specific expression of calcium sensor gene *AmCBL1* in *Ammopiptanthus mongolicus* was found to be induced by multiple stresses, and various plant hormones at different time points (Guo et al., 2010).

Use of multiple motif-finding algorithms in parallel provided robust result in terms of discovering globally over-represented signature motifs in the phloem-specific promoters. The A/N-rich motifs, identified by AlignACE (Figure S3), were conspicuous and non-specific. Similar kind of A/T rich motif was over-represented in pumpkin promoters of phloem-specific transctipts (Ruiz-Medrano et al., 2011). MEME and RSAT info-gibbs programs identified GA/CT repeat motifs, over-represented in the phloem-specific promoter sequences. Similar GA/CT and CT/GA repeats were also identified in promoter sets of phloem transcripts in pumpkin, cucumber, poplar, and rice (Ruiz-Medrano et al., 2011). It was proved that the motifs with CT/GA-repeat signature could drive vascular-specific gene expression when they were fused with the minimal promoter sequences in Arabidopsis (Ruiz-Medrano et al., 2011). In PLACE database GA/CT-rich motifs showed best match with GAGA8HVBKN3 motif, which is a GA octa-dinucleotide repeat found in intron IV of barley (H.v.) Bkn3 gene and bound by a nuclear protein, Barley B recombinant (BBR) (Santi et al., 2003). In spite of variable number of *cis*- elements across the phloem-specific promoters any correlation between the dosage of *cis*-elements and the promoter-strength was not apparent (Figure 5B). Therefore, the likely *cis*-regulatory logic governing the expression pattern and strength of the promoters lied in the combinatorial interaction among the set of DNA-elements in and around the promoter (Zou et al., 2011). GS3A promoter which showed strikingly higher activity compared to the other promoters, uniquely contained the degenerate motif–GRRRGGGAGASG-(*R* = A/G, *S* = G/C), which showed functional homology to GA octadinucleotide repeat of barley Bkn3 gene as evident by lowest *E*-value in pair-wise comparison.

In most of the studies comparing different phloem-specific promoters for transgene expression (Dutt et al., 2012; Benyon et al., 2013; Miyata et al., 2013) the assay for expression has been carried out at a particular developmental stage of the transgenic plants. However, for expressing transgenic resistance against insects like aphids, trait-expression across the plant developmental stages is pivotal. Therefore, a RT-qPCR based statistical method employing four algorithms has been used for analysing temporal stability of the seven *B. juncea* promoters. geNorm and NormFinder are mostly used for analysing expression-consistency of the reference genes in RT-qPCR analysis (Chandna et al., 2012; Ling et al., 2014). The analyses empirically demonstrated differential influence of plant growth-stage on the promoter-activities and relatively higher promoter-stability of the six phloem-bound promoters, except GAS1.

## Conclusion

Our study identified the conserved DNA-motifs that presumably define the phloem-specificity of gene-expression. However, the complex task of fully understanding architecture of the phloem-specific promoters will essentially require complementation based functional validation of the identified signature-motifs, and particularly their interaction in combinatorial control of gene expression. Nevertheless, the signature-motifs identified in this study can be used for quick identification of potential phloem-specific promoters across the species based on the available sequence data. Also, for the first time MS Excel based statistical algorithms have been used for measuring expression-stability of the promoters based on gene-expression data of their cognate genes. In future perspective, it will be intriguing to examine whether complementation of the phloem-specific motifs to a promoter can endow phloem-specific activity to it.

## Author contributions

RB, MK: Conceived the idea, designed the experiments and wrote the manuscript; MK, DB: Performed the experiments and analyzed the data; MN, PJ, and PV: contributed reagents/analysis tools/logistics.

### Conflict of interest statement

The authors declare that the research was conducted in the absence of any commercial or financial relationships that could be construed as a potential conflict of interest.

## Supplementary material

Figure S1 **RNA quality and validation of amplification-specificity in RT-qPCR. (A)** RNA gel visualized under UV. **(B)** Desired PCR amplification viewed in 2% agarose gel along with 100 bp DNA ladder M. **(C)** Melt-curves showing single peak generated in RT-qPCR. (TIF).

Figure S2 **Amplification efficiency of the gene-specific primer sets used in qRT-PCR**.

Figure S3 **Consensus nucleotide sequences identified in plant phloem-specific promoters using the AlignACE program**. The *X*- and *Y*-axis show the position of nucleotides and the bits score, respectively. Two motifs with highest score are shown.

Table S1 **Primer sequences and related information of the genes used in RT-qPCR**.

Table S2 **List of primer sequences and related information of the genes used in RT-PCR**.

Table S3 **Phloem-specific promoters from different plant species**.

## Abbreviations

RT-PCR: Reverse transcription polymerase chain reaction
RT-qPCR: Reverse transcription quantitative real time PCR
MJ: Methyl jasmonate
SA: Salicylic acid
ET: Ethylene
ABA: Abscisic acid
GA: Gibberellic acid.
