# Supplementary figures and images for: Strength, Stability, and cis-Motifs of In silico Identified Phloem-Specific Promoters in Brassica juncea (L.)
Source: Front Plant Sci. 2016 Apr 18;7:457. doi: 10.3389/fpls.2016.00457 (PMC4834444; doi:10.3389/fpls.2016.00457)

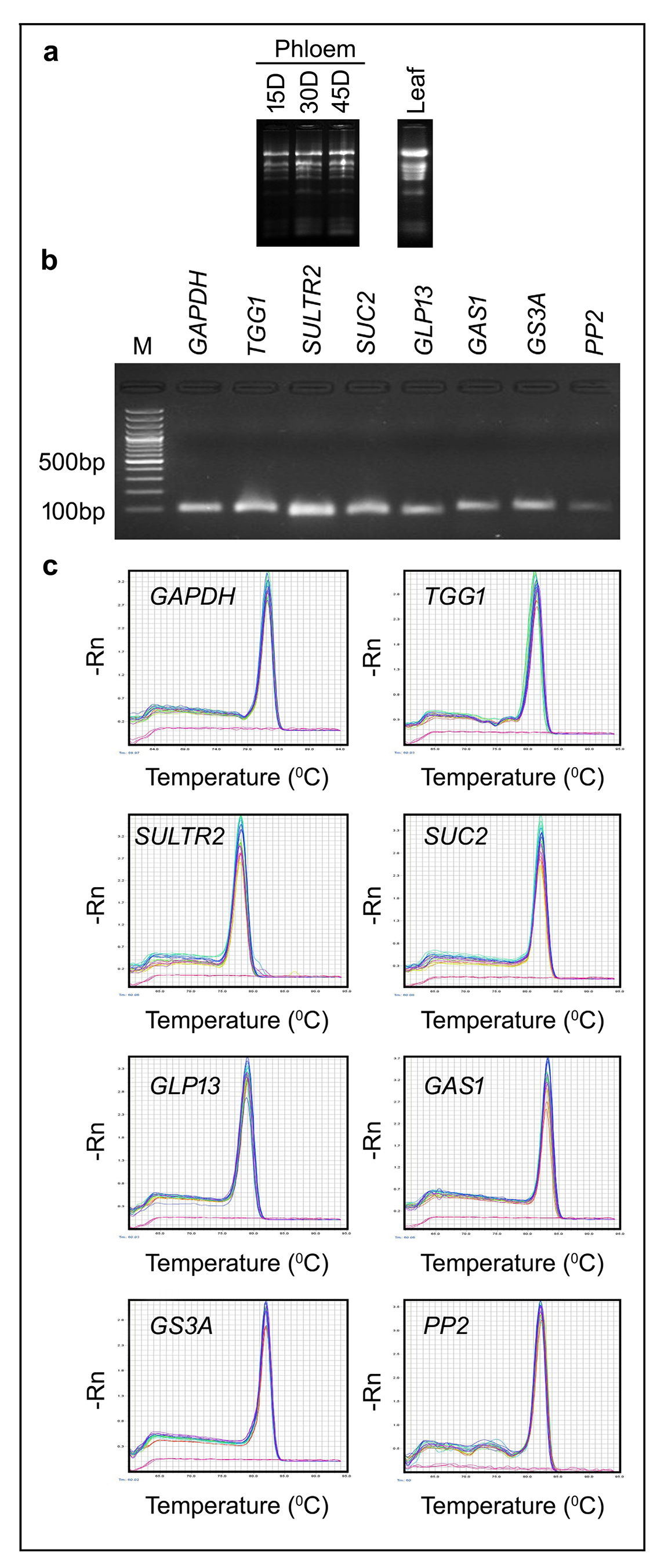

Supplement: Figure S1 — RNA quality and validation of amplification-specificity in RT-qPCR. (A) RNA gel visualized under UV. (B) Desired PCR amplification viewed in 2% agarose gel along with 100 bp DNA ladder M. (C) Melt-curves showing single peak generated in RT-qPCR. (TIF). [file Image1.TIF]

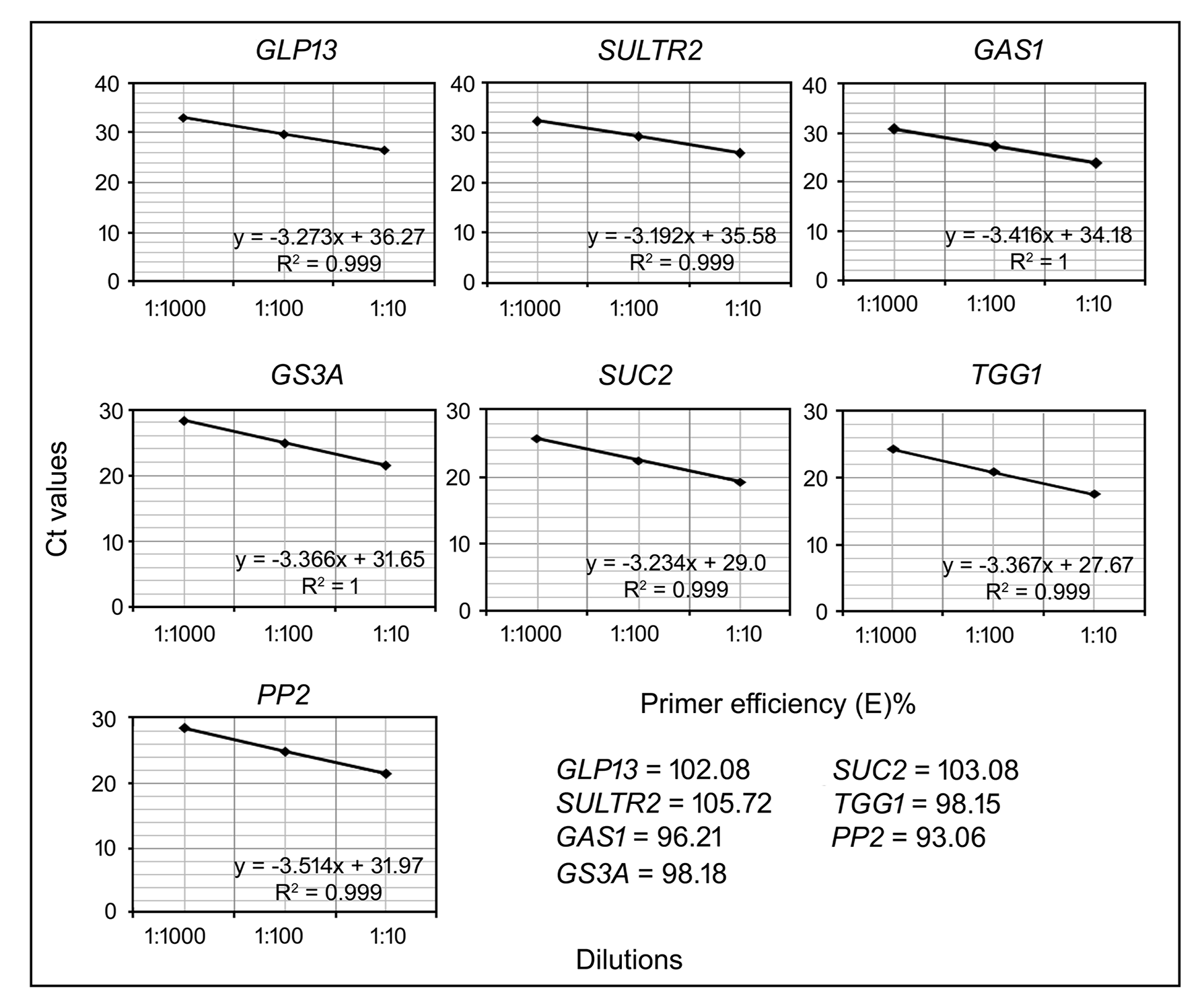

Supplement: Figure S2 — Amplification efficiency of the gene-specific primer sets used in qRT-PCR. [file Image2.TIF]

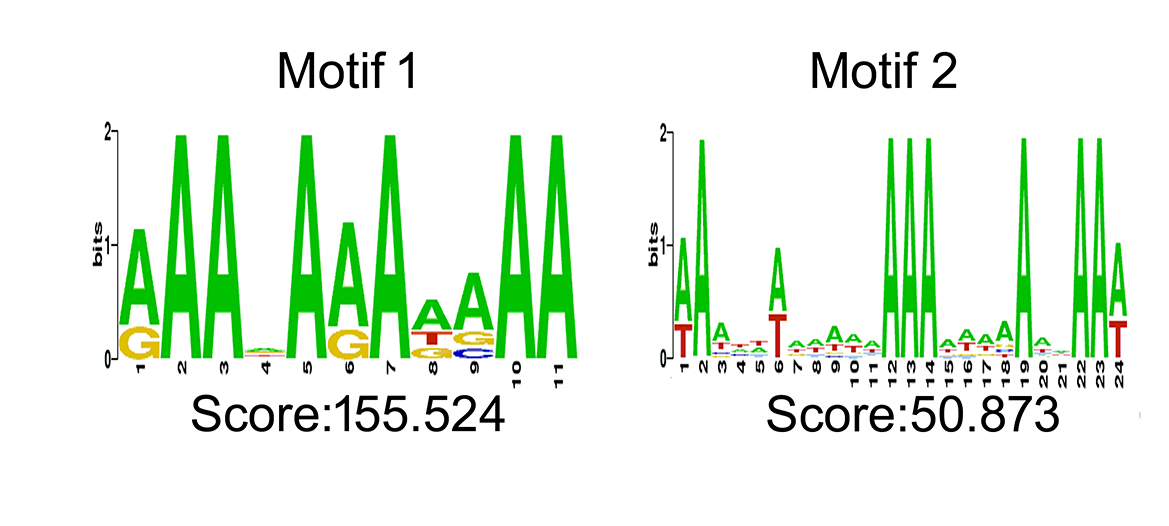

Supplement: Figure S3 — Consensus nucleotide sequences identified in plant phloem-specific promoters using the AlignACE program. The X- and Y-axis show the position of nucleotides and the bits score, respectively. Two motifs with highest score are shown. [file Image3.TIF]
