# Supplementary material for: Strength, Stability, and cis-Motifs of In silico Identified Phloem-Specific Promoters in Brassica juncea (L.)
Source: Front Plant Sci. 2016 Apr 18;7:457. doi: 10.3389/fpls.2016.00457 (PMC4834444; doi:10.3389/fpls.2016.00457)
Supplement: Table S1 — Primer sequences and related information of the genes used in RT-qPCR. [file Table1.DOCX]

**Table S1.** **Primer sequences and related information of the genes** **used in RT-qPCR**

| **Gene** | **GenBank**  **Acc no#** | **Spices** | **Primer (F/R; 5’🡪3’)** | **Amplicon length**  **( bp)** | **Tm**  **(^0^ C)** | **Amplification**  **Efficiency**  **(%)** | **Regression**  **coefficient (R^2^)** |
| --- | --- | --- | --- | --- | --- | --- | --- |
| *GLP13* | AC189540.1 | *Brassica rapa* | TACACCATCATCTCTCCCTCTT  TGGTTAAAGGACGGGTTTACTC | 88 | 62 | 102.0 | 0.999 |
| *GSA3* | AY773089.1 | *Brassica rapa* | TTGCTGGTGTGGTTGTATCT  TATCCTCCTTCCTCCCTCATC | 109 | 62 | 98.1 | 1.000 |
| *TGG1* | AY014960.1 | *Brassica juncea* | CTCGACGACAGAAACCTCAAA AGGCTTGAACGGAGGAAATC | 101 | 62 | 98.1 | 0.999 |
| *GAS1* | FJ407183.1 | *Brassica napus* | CCGCCGTGACGATAACTAAA  CTAAGCCTACGACTCCTTTGAC | 105 | 62 | 96.2 | 1.000 |
| *SUC 2* | EU570076.1 | *Brassica napus* | TACAGTTCGGTTGGGCTTTAC  CCACAGAGCCAAATCAGAGAA | 97 | 62 | 103.8 | 0.999 |
| *SULTR2* | AJ223495.1 | *Brassica juncea* | GAGATGGAGGGTACTTCACAAA  CGGCTTCTCCTACTGTCATAAA | 89 | 62 | 105.6 | 0.999 |
| *PP2* | XM_009110192 | *Brassica rapa* | GATGTCAGAGTTGGCGAGTT  GGAGAGCCCTTTCTTCCAAA | 100 | 62 | 93.0 | 0.999 |
| *BjGAPDH* | AF536826.1 | *Brassica rapa* | CTAACTGCCTTGCTCCACTT  TGTCTTCTGAGTTGCAGTGATAG | 101 | 62 | * | * |

* Used as an internal control in qRT-PCR
