# Supplementary material for: Strength, Stability, and cis-Motifs of In silico Identified Phloem-Specific Promoters in Brassica juncea (L.)
Source: Front Plant Sci. 2016 Apr 18;7:457. doi: 10.3389/fpls.2016.00457 (PMC4834444; doi:10.3389/fpls.2016.00457)
Supplement: Table S2 — List of primer sequences and related information of the genes used in RT-PCR. [file Table2.DOCX]

**Table S2. List of primer sequences and related information of the genes used in RT-PCR**

| **Gene** | **GenBank**  **Acc no#** | **spices** | **Primer (F/R; 5’🡪3’)** | **Amplicon length**  **( bp)** | **Tm**  **(^0^ C)** |
| --- | --- | --- | --- | --- | --- |
| *RbcS* | HQ404361.1 | *Brassica juncea* | AGCAACGGAGGAAGAGTTAG  CCAGTAACGTCCATCGTAGTATC | 226 | 62 |
| *Lhca2* | EF471212.2 | *Brassica juncea* | GCCGGAGAGCAAGAGTATTT  ACCCATCACTGCCAACATAG | 285 | 62 |
| *UBC9* | XM_009110868.1 | *Brassica rapa* | CTTACTCTGGTGGTGTGTTTCT  CAGTGGACTCGTACTTGTTCTT | 282 | 62 |
