# Supplementary material for: Strength, Stability, and cis-Motifs of In silico Identified Phloem-Specific Promoters in Brassica juncea (L.)
Source: Front Plant Sci. 2016 Apr 18;7:457. doi: 10.3389/fpls.2016.00457 (PMC4834444; doi:10.3389/fpls.2016.00457)
Supplement: Table S3 — Phloem-specific promoters from different plant species. [file Table3.docx]

**Table S3. Phloem-specific promoters from different plant species**

| **S.N.** | **Name of the genes** | **Promoter** | **Promoter size (kb)** | **Source** | **References** |
| --- | --- | --- | --- | --- | --- |
| 1 | Sucrose Synthase | AtSUS1 | 1.5 | *Arabidopsis thaliana* | Martin et al. 1993 |
| 2 | Germin like protein | AtGLP13 | 0.762 | *Arabidopsis thaliana* | Yang et al. 2013 |
| 3 | Sucrose H^+^ symporter | AtSUC2 | 0.941 | *Arabidopsis thaliana* | Truernit and Sauer 1995 |
| 4 | Sucrose Synthase | OsRSs1 | 1.954 | *Oryza sativa* | Shi et al. 1994 |
| 5 | Phloem protein | OsRpp16 | 1.346 | *Oryza sativa* | Asnao et al. 2002 |
| 6 | Phloem protein | OsRpp17 | 2.0 | *Oryza sativa* | Asnao et al. 2002 |
| 7 | Sucrose synthase 1 | ZmSh1 | 0.804 | *Zea mays* | Yang and Russell 1990 |
| 8 | Phloem protein 2 | CmPP2 | 1.115 | *Cucurbita moschata* | Guo et al. 2004 |
| 9 | Nodule autoregulation receptor kinase | GmNRK | 1.7 | *Glycine max* | Nontachaiyapoom et al. 2007 |
| 10 | Invertase | StinvCD111 | 0.922 | *Solanum tuberosum* | Hedley et al. 2000 |
| 11 | Invertase | StinvCD141 | 1.424 | *Solanum tuberosum* | Hedley et al. 2000 |
| 12 | Galactinol synthase | CmGAS1 | 3.0 | *Cucumis melo* | Haritatos et al. 2000 |
| 13 | Sucrose synthase 1 | CsSUS1-1 | 1.652 | *Citrus sinensis* | Singer et al. 2011 |
| 14 | Sucrose synthase 1 | CsSUS1-2 | 1.704 | *Citrus sinensis* | Singer et al. 2011 |
| 15 | Calcium sensor | AmCBL1 | 1.683 | *Ammopiptanthus mongolicus* | Guo et al.2010 |
| 16 | Phloem protein 2 | AtPP2-A1 | 0.98 | *Arabidopsis thaliana* | Miyata et al. 2013 |
| 17 | 5-Methylthioribose kinase1 | AtMTK1 | 1.220 | *Arabidopsis thaliana* | Pommerrenig et al. 2011 |
| 18 | Acidoreductone oxygenase 1 | AtARD 1 | 1.638 | *Arabidopsis thaliana* | Pommerrenig et al. 2011 |
| 19 | Acidoreductone oxygenase 2 | AtARD2 | 1.230 | *Arabidopsis thaliana* | Pommerrenig et al. 2011 |
| 20 | Acidoreductone oxygenase3 | AtARD3 | 1.617 | *Arabidopsis thaliana* | Pommerrenig et al. 2011 |
| 21 | Acidoreductone oxygenase4 | AtARD4 | 1.271 | *Arabidopsis thaliana* | Pommerrenig et al. 2011 |
| 22 | 5-Methylthioadenosine nucleosidase | AtMTN1 | 1.321 | *Arabidopsis thaliana* | Pommerrenig et al. 2011 |
| 23 | 5-Methylthioribose-1-phosphate isomerase1 | AtMTI | 1.925 | *Arabidopsis thaliana* | Pommerrenig et al. 2011 |
| 24 | Thioglucoside glucohydrolase 1 | AtTGG1 | 2.628 | *Arabidopsis thaliana* | Husebye et al. 2002 |
| 25 | Glutamine synthetase | PsGS3A | 1.92 | *Pisum sativum* | Brears et al. 1991 |
| 26 | CLV1-like leucine-rich repeat receptor kinase | MtSUNN | 1.36 | *Medicago truncatula* | Schnabel et al. 2012 |
| 27 | Sulphate transporter | AtSULTR2 | 2.0 | *Arabidopsis thaliana* | Takahashi et al. 2000 |
| 28 | Phosphatidylinositol-4-phosphate 5-kinase | AtPPK | 1.5 | *Arabidopsis thaliana* | Ruiz-Medrano et al. 2011 |
| 29 | bZIP family transcription factor (TGA1) | AtTGA1 | 2.668 | *Arabidopsis thaliana* | Ruiz-Medrano et al. 2011 |
| 30 | Actin depolymerizing factor 3 | AtADF3 | 1.651 | *Arabidopsis thaliana* | Ruiz-Medrano et al. 2011 |
| 31 | APPR2 pseudoresponse regulator | AtAPPR2 | 2.211 | *Arabidopsis thaliana* | Ruiz-Medrano et al. 2011 |
| 32 | ECT2 | AtECT2 | 1.781 | *Arabidopsis thaliana* | Ruiz-Medrano et al. 2011 |
| 33 | Rho guanyl-nucleotide exchange factor | AtRhoGEF | 2.103 | *Arabidopsis thaliana* | Ruiz-Medrano et al. 2011 |
| 34 | Leucine-rich repeat transmembrane protein kinase | AtLRRRK | 2.102 | *Arabidopsis thaliana* | Ruiz-Medrano et al. 2011 |
| 35 | ARF19 (Auxin Response factor 19) | AtARF19 | 1.718 | *Arabidopsis thaliana* | Ruiz-Medrano et al. 2011 |
| 36 | WRKY19 | AtWRKY19 | 1.5 | *Arabidopsis thaliana* | Ruiz-Medrano et al. 2011 |
| 37 | Glutaredoxin family protein | AtGRXFP | 1.717 | *Arabidopsis thaliana* | Ruiz-Medrano et al. 2011 |
| 38 | NAC domain-containing protein | AtANAC075 | 1.5 | *Arabidopsis thaliana* | Ruiz-Medrano et al. 2011 |
| 39 | NAC domain-containing protein | AtANAC100 | 1.708 | *Arabidopsis thaliana* | Ruiz-Medrano et al. 2011 |
